# MS Sunshine Study: Sun Exposure But Not Vitamin D Is Associated with Multiple Sclerosis Risk in Blacks and Hispanics

**DOI:** 10.3390/nu10030268

**Published:** 2018-02-27

**Authors:** Annette Langer-Gould, Robyn Lucas, Anny H. Xiang, Lie H. Chen, Jun Wu, Edlin Gonzalez, Samantha Haraszti, Jessica B. Smith, Hong Quach, Lisa F. Barcellos

**Affiliations:** 1 Los Angeles Medical Center, Department of Neurology, Southern California Permanente Medical Group, 100 S Los Robles, Pasadena, CA 91101, USA; 2 College of Medicine, Biology & Environment, Australian National University, Canberra, ACT 2000, Australia; robyn.lucas@anu.edu.au; 3 Department of Research & Evaluation, Kaiser Permanente Southern California, 100 S. Los Robles Avenue, Pasadena, CA 91101, USA; anny.h.xiang@kp.org (A.H.X.); lie.h.chen@kp.org (L.H.C.); jun.x.wu@kp.org (J.W.); edlin.g.gonzales@kp.org (E.G.); samanthaha@pcom.edu (S.H.); Jessica.b.smith@kp.org (J.B.S.); 4 Philadelphia College of Osteopathic Medicine, 4170 City Avenue, Philadelphia, PA 19131, USA; 5 QB3 Genetic Epidemiology and Genomics Lab, School of Public Health, University of California Berkeley, 209 Hildebrand Hall, Berkeley, CA 94720-7356, USA; hquach@berkeley.edu (H.Q.); lbarcellos@berkeley.edu (L.F.B.)

**Keywords:** multiple sclerosis, vitamin D, sun exposure, blacks, Hispanics

## Abstract

Multiple sclerosis (MS) incidence and serum 25-hydroxyvitamin D (25OHD) levels vary by race/ethnicity. We examined the consistency of beneficial effects of 25OHD and/or sun exposure for MS risk across multiple racial/ethnic groups. We recruited incident MS cases and controls (blacks 116 cases/131 controls; Hispanics 183/197; whites 247/267) from the membership of Kaiser Permanente Southern California into the MS Sunshine Study to simultaneously examine sun exposure and 25OHD, accounting for genetic ancestry and other factors. Higher lifetime ultraviolet radiation exposure (a rigorous measure of sun exposure) was associated with a lower risk of MS independent of serum 25OHD levels in blacks (adjusted OR = 0.53, 95% CI = 0.31–0.83; *p* = 0.007) and whites (OR = 0.68, 95% CI = 0.48–0.94; *p* = 0.020) with a similar magnitude of effect that did not reach statistical significance in Hispanics (OR = 0.66, 95% CI = 0.42–1.04; *p* = 0.071). Higher serum 25OHD levels were associated with a lower risk of MS only in whites. No association was found in Hispanics or blacks regardless of how 25OHD was modeled. Lifetime sun exposure appears to reduce the risk of MS regardless of race/ethnicity. In contrast, serum 25OHD levels are not associated with MS risk in blacks or Hispanics. Our findings challenge the biological plausibility of vitamin D deficiency as causal for MS and call into question the targeting of specific serum 25OHD levels to achieve health benefits, particularly in blacks and Hispanics.

## 1. Introduction

Evidence from animal models and observational studies [1,2,3] in white populations shows that higher levels of serum 25-hydroxyvitamin D (25OHD) are associated with reduced risk of multiple sclerosis (MS). Some consider that causality has been established and vitamin D supplementation to prevent MS is widely supported [4]. 

This interpretation of the evidence is not universal. The 2011 systematic review of the Institute of Medicine noted that “Taken together, these observational studies show widely variable outcomes for associations between serum 25OHD levels and MS… lack of causal evidence further diminishes the likelihood for a relationship between vitamin D and MS” [5]. 

If there is a causal biological link between vitamin D and MS, it would be expected to be present across different racial/ethnic groups. Available studies show blacks and Hispanics have lower 25OHD levels than whites [6] yet the incidence of MS is lower in Hispanics [7] and similar in blacks compared to whites [7,8]. In addition, there is substantial evidence that while true 25OHD deficiency leads to rickets in all racial/ethnic groups, ‘insufficient’ 25OHD levels in blacks do *not* lead to increased risk of fractures or decline in bone mineral density [9,10]. 

The vitamin D-MS hypothesis originated from the observation that the prevalence of MS increases with increasing distance from the Equator, as ultraviolet radiation (UVR) from the sun [11] becomes less intense—but also, traditionally, where more white people live. Exposure to UVR is, in turn, the principal natural source of vitamin D by stimulating intradermal synthesis; thus, low UVR (further from the Equator) should lead to low vitamin D status, and a possible explanation for the geographic distribution of MS. This relationship between UVR exposure and 25OHD levels is very strong in light skinned individuals. However, people with darker skin tones produce less 25OHD following the same amount of UVR exposure as whites. Furthermore, more evidence is emerging that UVR has a plethora of immunological effects independent of the vitamin D pathway [12] that are not attenuated by darker skin tone [13].

The only study in whites to simultaneously examine sun exposure and 25OHD showed that 25OHD levels alone do not explain the protective association between lifetime sun exposure and MS risk [2]. Whether sun exposure or 25OHD is associated with MS risk in blacks and Hispanics is unknown. The only previous study in non-whites found no association between serum 25OHD levels and the risk of MS in blacks [1], but it did not measure sun exposure.

The primary purpose of the MS Sunshine study was to test the vitamin D-MS hypothesis in blacks and Hispanics. Because the efficiency of intradermal vitamin D synthesis varies by race/ethnicity, studying blacks, Hispanics and whites provides a natural experiment for testing the potential protective effects of 25OHD levels on MS. This trans-ethnic population also provides a unique opportunity to test whether higher lifetime sun exposure is potentially protective independent of adult 25OHD levels, as the non-vitamin D immunosuppressive effects of UVR are less affected by skin color. 

## 2. Materials and Methods

### 2.1. Study Population

Participants in the MS Sunshine Study were recruited from the Kaiser Permanente Southern California (KPSC) membership between December 2011 and May 2015 via mailings and telephone contact. KPSC is a large prepaid health maintenance organization with over 4 million members representative of the general population in Southern California [14]. KPSC uses an integrated electronic health record (EHR) system which includes all inpatient and outpatient encounters, diagnostic tests, diagnoses and medications as well as some demographic and behavioral characteristics. Data were collected from the EHR and after informed consent, structured in-person interview and blood was drawn for 25OHD measurement (if not available in EHR) and genotyping.

#### 2.1.1. Standard Protocol Approvals, Registrations, and Patient Consents

All subjects gave their informed consent for inclusion before they participated in the study. The study was conducted in accordance with the Declaration of Helsinki, and the protocol was approved by the KPSC institutional review board (IRB 5962).

#### 2.1.2. Case Identification

Incident cases with MS or clinically isolated syndrome (CIS) were identified using similar methods as previously described [7,15,16]. Briefly, we searched EHRs monthly for first mention of diagnostic codes for MS or CIS and diagnoses were confirmed by an MS specialist (ALG) according to diagnostic criteria [17,18,19]. Eligibility required diagnosis of MS or CIS within the past 1.5 years or symptom onset within the past 3 years, and age ≥ 18 years (see Appendix A).

#### 2.1.3. Control Selection

Once a case interview was completed, at least 1 control participant from the KPSC population, matched to the case on race/ethnicity, date of birth (within 2 years), sex and home KPSC facility (a surrogate measure for socioeconomic status) was identified from the EHR and recruited. The controls were assigned the same index date as their matched case (symptom onset date). For participation rates and additional details, see Appendix A.

### 2.2. Data Collection

Self-identified race/ethnicity was obtained from the interview. White, non-Hispanics were classified as white; any black race regardless of ethnicity was classified as black; and those who identified themselves as white, Hispanics were classified as Hispanics. Comparison with genetic ancestry markers validated the accuracy of self–identified race/ethnicity.

Covariates obtained from the interview included sun exposure during weekends and holidays (leisure time) age 6 years to date of interview, places of residence since birth, smoking (never/ever) and vitamin D supplement use defined as ≥600 IU daily at or within 3 months prior to 25OHD measurement. Age was defined as age at index date. Body mass index (BMI) closest to the date of 25OHD measurement was obtained from EHR. 

Total serum 25OHD was measured using liquid chromatography, tandem mass spectrometry. The sensitivity of the assay is <2.5 nmol/L. The intra-and inter-assay coefficients of variation are less than 5.2% at 25, 62.5 and 192.5 nmol/L.

### 2.3. Genotyping

HLA-DRB1*15:01 status, the major risk allele for MS, was determined using a tag SNP (rs3135388). Genetic ancestry was determined with the software STRUCTURE Version 2.3.1, University of Oxford, Oxford, UK [20] to infer the presence of distinct populations. A genome–wide set of 67547 linkage disequilibrium pruned loci were selected using PLINK 1.07, Center for Human Genetic Research, the Broad Institute of Harvard & MIT, Boston, USA [21]. We compared the structure outputs from three (Europeans, Africans, and Amerindians), five (Europeans, Africans, Amerindians, East Asians, and Central/South Asians), and seven (Europeans, Africans, Amerindians, East Asians, Central/South Asians, Western Asians and Oceanians) reference populations, and concluded that using 5 or 7 reference populations did not improve upon the three-population model for estimating population admixture in our cohort. With three populations assumed, the probability of population ancestry was estimated by specifying a 10,000 iteration burn-in period and a 10,000 iteration follow-up of the Markov Chain Monte Carlo model utilized by STRUCTURE. 

### 2.4. Statistical Analysis

#### 2.4.1. Sun Exposure

We used the most rigorous method available for assessing lifetime sun exposure. Specifically, we calculated cumulative lifetime UVR for each participant by combining latitude of residence and usual time outdoors obtained from a detailed residency calendar with ambient ultraviolet radiation levels obtained from satellite-derived ground level estimates [22]. The questionnaire we used has been validated both for recall validity [23] and for external validity against an objective measure of lifetime sun exposure, silicone skin casts for actinic damage in whites [24]. The average monthly UVR estimates for each participant were summed from age 6 years to symptom onset/index date.

#### 2.4.2. Recent Sun Exposure and 25OHD

Multivariable linear regression was used to examine the association of UVR during summer and winter in the 12 months prior to symptom onset/index, age, sex, BMI and season of blood draw on 25OHD levels (dependent variable; log transformed).

#### 2.4.3. Lifetime Sun Exposure, 25OHD and MS

Multivariable unconditional logistic regression was used to simultaneously estimate the independent odds ratios (OR) and 95% confidence intervals (CI) of 25OHD and cumulative lifetime UVR (KJ/m^2^) on MS/CIS by race/ethnicity. 25OHD was log-transformed (normal distribution). Both cases’ and controls’ 25OHD values were deseasonalized by using residuals derived from multivariable linear regression to control for the season of blood draw (April–September or October–March) and adjusted for BMI because BMI had a strong association with 25OHD levels but not MS/CIS risk. Models testing the association between UVR exposure, 25OHD and risk of MS/CIS were adjusted for age, sex, genetic ancestry, smoking and HLA-DRB1*15:01 carrier status. 

#### 2.4.4. 25OHD and MS

Several additional approaches were used to try and detect an association between 25OHD and MS/CIS in blacks or Hispanics. 25OHD was modeled as both raw and deseasonalized values in the following ways: continuous; continuous in the log transformation; according to the cut–points recommended by the Institute of Medicine (30, 50 nmol/L) and the US Endocrine Society (75 nmol/L) [25]; and in quintiles. To allow for direct comparison with previous studies [1,2,3], conditional logistic regression was also used to estimate the matched OR and its corresponding 95% CI for the association between MS/CIS and deseasonalized 25OHD within each racial/ethnic category (white, black or Hispanic). For these analyses, 25OHD values were deseasonalized by fitting a polynomial regression in controls within each race/ethnicity [2] (see Appendix A).

Sensitivity analyses excluding vitamin D supplement users were conducted for all analyses. Two-sample *t*-tests were used to compare means, Wilcoxon-Mann-Whitney test for non-normally distributed variables and χ^2^ or Fisher exact test to compare frequencies between two groups. All analyses were conducted using SAS software v9.3 (SAS Institute, Cary, NC, USA).

## 3. Results

### 3.1. Characteristics of Participants

Table 1 shows the demographic characteristics, prevalence of MS risk factors and selected factors that influence 25OHD levels in cases and controls. There was a stronger female preponderance among blacks than whites (*p* < 0.001). Hispanics were younger at symptom onset than whites or blacks (*p* < 0.001). Whites were more likely to be current or former cigarette smokers (*p* < 0.001) and had lower BMI (*p* < 0.001) than blacks and Hispanics. The median time from symptom onset to diagnosis was slightly longer among black cases (six months; interquartile range [IQR] 1–22 months) than Hispanics (three months, IQR 0–15) and whites (four months, IQR 1–19). 

### 3.2. Factors Associated with 25OHD Levels

UVR exposure in the 12 months prior to the index date in whites (*β* = 0.0025, *p* < 0.001) and Hispanics (*β* = 0.0022, *p* = 0.025) but not in blacks (*β* = 0.0013, *p* = 0.24) was independently associated with 25OHD levels. Higher BMI was strongly associated with lower 25OHD in whites (*p* < 0.0001), Hispanics (*p* = 0.0002) and blacks (*p* = 0.0006). Independent effects of season on 25OHD levels were also observed in these models (*p* = 0.04, <0.0001 and 0.006 in whites, Hispanics and blacks, respectively).

### 3.3. Cumulative Lifetime UVR, 25OHD and MS

**Figure 1:**
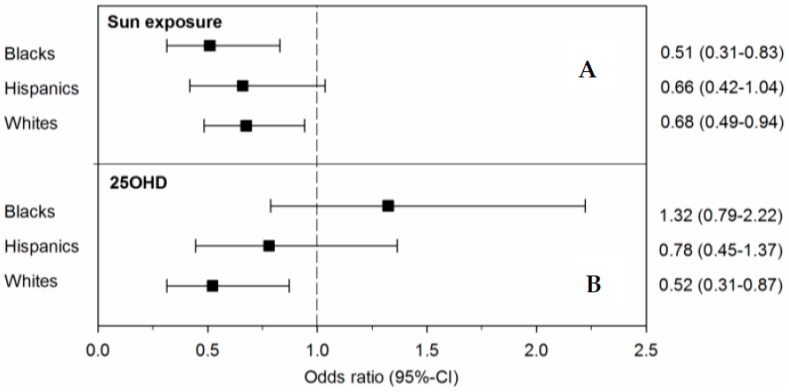
Simultaneous effects of lifetime ultraviolet radiation exposure and 25-hydroxyvitamin D on MS risk. Depicted are the mutually adjusted odds ratios (OR) and 95% confidence intervals (95% CI) of the independent associations of lifetime ultraviolet radiation exposure (Panel A) or serum 25-hydroxyvitamin D levels (Panel B) with risk of multiple sclerosis/clinically isolated syndrome within each racial/ethnic group obtained from the same model. Odds ratios depicted are adjusted by age, sex, smoking, genetic ancestry, HLA–DRB1*15:01(rs3135388) 25-hydroxyvitamin D (Panel A) or lifetime ultraviolet radiation exposure (Panel B). Serum 25-hydroxyvitamin D levels were log transformed, adjusted for BMI and deseasonalized.

Cumulative lifetime UVR was lower among cases compared to controls in all 3 groups (Table 2). The protective association between higher lifetime UVR exposure and a lower risk of MS persisted after accounting for 25OHD in blacks (*p* = 0.007) and whites (*p* = 0.020) (Figure 1) although it did not reach statistical significance in Hispanics (*p* = 0.071). 

Serum 25OHD levels were higher among whites than blacks, and Hispanics had intermediate values (Table 2). Serum 25OHD levels were significantly lower among white cases than controls but there was no significant difference in 25OHD levels between black or Hispanic cases and their respective controls (Table 2). The median time from diagnosis to 25OHD measurement was somewhat shorter among black cases (1 month, IQR-1–9), than Hispanics (4 months, IQR 0–12) and whites (4 months, IQR 0–10).

### 3.4. 25OHD and MS

Regardless of how 25OHD was defined, models either adjusted or unadjusted, raw, deseasonalized and matched deseasonalized, an association between high 25OHD and a significantly lower odds ratio of MS/CIS was found only in whites (*p* = 0.013; Figure 1; Table 2 and Table 3). In blacks, 25OHD levels > 75.0 nmol/L were associated with increased risk of MS although this did not reach statistical significance (Table 3). Use of vitamin D supplements at the time of 25OHD measurement was more common among cases than controls and more common among whites and blacks compared with Hispanics (Table 1). Excluding supplement users did not significantly alter the results (data not shown).

## 4. Discussion

Our findings demonstrate that there is a strong and consistent association between higher lifetime sun exposure and MS risk across racial/ethnic groups. However, the protective association of sun exposure is not explained by current serum 25OHD levels in blacks, Hispanics or even fully in whites. This indicates that the protective effect of sun exposure is most likely mediated through immunomodulatory mechanisms independent of vitamin D. Taken together with previous data from multi-ethnic studies of determinants of vitamin D status [6,26,27], these findings call into question the common practice of targeting a specific serum 25OHD level with the expectation of health benefits, particularly in blacks and Hispanics.

We looked very carefully for any evidence of an association between 25OHD and MS in non-white populations, in view of the purported causal association between vitamin D deficiency and increased risk of MS [4]. We found evidence to support an association between higher 25OHD level and reduced MS/CIS risk among whites, but no evidence of any protective effect in Hispanics and blacks. Potential bias introduced by case-control design should be in the direction of finding such an association (as we and others found in whites), as MS patients, particularly those with disability, often avoid sun exposure because heat makes them feel worse. Likewise, multiple statistical tests would be expected to lead to false positive results rather than consistently negative findings.

If there is a causal biological link between vitamin D and MS, it would be expected to be present across different racial/ethnic groups. Randomized controlled trials to prove such causality for the onset of MS are not feasible; this requires innovative approaches to rigorously test the hypothesis. Mendelian randomization has been used in an attempt to establish causality between vitamin D and MS in whites [28,29] but it is unclear if the key assumptions required for this approach to be valid have been met. We believe that our findings, from a rigorously conducted observational study, with detailed measurement of both sun exposure and 25OHD levels (available in very few studies), and across different races (available only in this study) provides the strongest test to date of the vitamin D-MS hypothesis. 

These findings are important because many experts are recommending vitamin D supplementation to prevent MS. In addition, the apparent association between low 25OHD and MS in whites has led to an explosion of vitamin D-MS studies while neglecting rigorous investigations of alternative hypotheses. 

Our findings suggest that while 25OHD may be an excellent surrogate measure of sun exposure in whites and certainly easier to measure, focusing on risk factors common to multiple racial/ethnic groups is more likely to lead to scientific advances.

While our finding that higher 25OHD is associated with a lower risk of MS in whites appears consistent with the 3 previous studies that have examined this question in adults [1,2,3], there are important discrepancies. A threshold effect was found in 2 studies but at different values (~100 [1] or ≥75 nmol/L [3]), a linear association in 1 study without a threshold effect [2]; and trends across quintiles in 2 studies [1,2]. We found a linear relationship only in log transformation (consistent with much larger population studies), and a positive association with MS in whites at all pre-determined threshold values and across quintiles. This is despite strikingly similar distributions of serum 25OHD levels in whites across these studies [1,2,3] and ours. 

Although it is possible that the differences that we see between racial/ethnic groups are due to some unknown genetic pathways, we favour a less complex explanation that the association between serum 25OHD and MS is found only in whites because it is a good surrogate measure of sun exposure in whites but not darker skinned individuals. Sun exposure is a major source of vitamin D synthesis by skin. Yet, the same amount of UVR exposure results in a smaller increase in 25OHD in dark-skinned compared to light-skinned groups [5]. Thus, it is possible that sun exposure is protective for MS but that serum 25OHD levels do not quantitatively capture this exposure in darker skinned individuals.

Our data are consistent with previous studies that examined environmental influences on serum 25OHD levels in blacks, Hispanics and whites [6,27,30,31]. We found a similarly strong correlation between recent UVR exposure and 25OHD levels in whites and Hispanics but less so in blacks [5]; and the expected effect of obesity and season on 25OHD levels in all 3 groups [6].

Our findings in whites are similar to those reported by the AusImmune Study [2] and a Swedish study [32], which both showed that the protective association of sun exposure is independent of the association between serum 25OHD and MS/CIS. 

That sun exposure could decrease the risk of MS through non–vitamin D dependent pathways is biologically plausible. UVR exposure results in immunomodulation through multiple mechanisms including generation of T regulatory cells, B suppressor (regulatory) cells and production of immunosuppressive lipid mediators and alarmins [33]. UVR has also been shown to suppress the animal model of MS (experimental autoimmune encephalomyelitis) through pathways independent of vitamin D [34,35,36]. 

An alternative explanation for our findings is that total serum 25OHD may not be a good reflection of the amount of bioavailable vitamin D—the form that is important in regulating the immune system. There is some evidence to support this in blacks who express a different dominant isoform of the vitamin D transporter protein (vitamin D-binding protein, DBP) than whites and Hispanics. The DBP found in most blacks is the most efficient transporter of 25OHD and its metabolites to target tissues [37] and is associated with higher bioavailable 25OHD levels. This may explain why, despite quite low total 25OHD levels, most blacks are not physiologically vitamin D deficient [38]. But this difference in bioavailable 25OHD levels would not explain why we did not find an association between 25OHD and MS in Hispanics whose DBP isoforms are like whites. This hypothesis is addressed in subsequent analyses from the MS Sunshine study [39].

Limitations of this study include the case-control design necessitating that most 25OHD measures were obtained after symptom onset (although very close to the time of diagnosis) and sun exposure was obtained retrospectively. MS often results in sun avoidant behavior due to heat sensitivity [40] which can cause an exaggerated association between low 25OHD and MS particularly in prevalent cases. This most likely explains the observed lower 25OHD levels in a previous study of prevalent black MS cases compared to controls [41] and may explain the strong association we found in whites, but does not explain the *lack* of association we found in Hispanics and blacks. Interestingly, 25OHD levels do not differ between prevalent Hispanic MS cases or controls [42], or decline with increasing disability as has been shown in whites [43]. Another limitation is that we relied on a single measure of 25OHD from adulthood. While a single measure is a reliable indicator of long term vitamin D status in adults [44] we cannot make any inference about the correlation with childhood/adolescent vitamin D status, which may be a critical risk period for the development of MS.

Similarly, recall bias of sun exposure would be expected to bias the results toward the null and does not explain the consistent protective association of UVR with MS seen across racial/ethnic groups. In addition to sun exposure, cumulative UVR, the most rigorous method available for estimating lifetime sun exposure, relies on places of residence and ambient UVR from geographic information services both of which are not subject to recall bias. 

During the course of this study, vitamin D supplement use after diagnosis also became popular but sensitivity analyses removing these participants did not significantly alter the findings. Selection bias, particularly among controls is another potential concern but comparison of age, smoking and BMI of participating and declined participants did not reveal significant differences. Women were more likely to participate regardless of case/control status. We also cannot exclude the possibility that the association between sun exposure and MS is a chance finding as a replication cohort of incident blacks or Hispanics with sun exposure and 25OHD measurements does not yet exist. However, the consistency between findings in blacks and Hispanics and with whites from the AusImmune Study [2] and Swedish study [32] is reassuring. Replication of our key findings should be addressed in future studies. 

This study highlights how multi–ethnic studies can lead to novel insights into disease. While we have been able to demonstrate a clear and consistent association with EBV [15] and sun exposure on MS risk across all 3 racial/ethnic groups, we are unable to find a similarly consistent association with cytomegalovirus infection [15] or serum 25OHD levels which strongly implies non–causal associations. Low serum 25OHD levels have been associated with multiple conditions and supplementing people with low 25OHD levels in the hopes of health benefits is popular. But results of randomized controlled trials have not demonstrated convincing health benefits of vitamin D supplementation [45,46]. Our findings suggest that time in the sun may be more beneficial than vitamin D supplementation for reducing the risk of MS, particularly in blacks and Hispanics.

## Figures and Tables

**Table 1:** Selected Characteristics of Study Participants at Index Date by Race/Ethnicity.

	Blacks (*n* = 247)			Hispanics (*n* = 380)			Whites (*n* = 514)		
	Cases (*n* = 116)	Controls (*n* = 131)	*p*-Value	Cases (*n* = 183)	Controls (*n* = 197)	*p*-Value	Cases (*n* = 247)	Controls (*n* = 267)	*p*-Value
Age, mean (SD), years	38.4 (12.8)	38.5 (13.0)	0.928	32.5 (10.7)	32.6 (11.1)	0.949	39.7 (12.0)	39.9 (12.2)	0.869
Female, *n* (%)	92 (79.3)	103 (78.6)	0.895	132 (72.1)	145 (73.6)	0.747	164 (66.4)	174 (65.2)	0.769
Smoking, *n* (%)	27 (23.3)	33 (25.2)	0.726	44 (24.0)	38 (19.3)	0.260	115 (46.6)	90 (33.7)	0.003
BMI, mean (SD)	30.5 (7.4)	31.6 (8.1)	0.260	29.5 (6.8)	30.0 (7.0)	0.423	28.5 (6.7)	28.5 (6.9)	0.948
VitD supplement users *, *n* (%)	28 (24.1)	2 (1.5)	<0.001	30 (16.4)	3 (1.5)	<0.001	62 (25.1)	19 (7.1)	<0.001
Season **, *n* (%)	56 (48.3)	65 (49.6)	0.833	85 (46.5)	104 (52.8)	0.217	116 (47.0)	142 (53.2)	0.159
HLA–DRB1*15:01, *n* (%)			0.20			0.0002			<0.0001
GG	95 (81.9)	115 (87.8)		134 (73.2)	174 (88.3)		136 (55.1)	214 (80.1)	
AG/AA	21 (18.1)	16 (12.2)		49 (26.8)	23 (11.7)		108 (44.9)	53 (19.9)	
% CIS	47.4%			56.8%			59.9%		

MS: multiple sclerosis; CIS: clinically isolated syndrome; SD: standard deviation; IQR: interquartile range; BMI: body mass index; VitD: vitamin D; *: at the time of 25OHD measurement; **: season (April–September) at the time of 25OHD measurement.

**Table 2:** Individual Relationships between Cumulative Ultraviolet Radiation Exposure or Serum 25-hydroxyvitamin D and Multiple Sclerosis.

	Cumulative UVR Dose *, 1000 KJ/m^2^				Serum 25-Hydroxyvitamin D, nmol/L		
	*n*	Mean (SD)	Adjusted OR (95% CI) **	*p*-Value	Median (IQR)	Adjusted OR (95% CI) ***	*p*-Value
**Blacks**							
cases	116	1.43 (0.77)	0.53 (0.32–0.85)	0.009	43.7 (32.4, 74.9)	1.21 (0.73–2.02)	0.455
controls	131	1.63 (0.79)			47.4 (30.0, 64.9)		
**Hispanic**							
cases	183	1.14 (0.61)	0.65 (0.41–1.01)	0.057	54.9 (42.4, 69.9)	0.74 (0.45–1.29)	0.290
controls	197	1.22 (0.68)			57.4 (44.9, 69.9)		
**Whites**							
cases	247	1.53 (0.79)	0.67 (0.48–0.93)	0.017	67.4 (49.9, 87.4)	0.52 (0.31–0.87)	0.010
controls	267	1.64 (0.83)			72.4 (59.9, 92.4)		

SD: standard deviation; IQR: interquartile range; UVR: ultraviolet radiation; OR: Odds Ratio; CI: confidence intervals; * from age 6 years to index date; ** adjusted for age, sex, smoking, genetic ancestry and HLA–DRB1*15:01; *** deaseasonalized log(25OHD) accounting for body mass index and adjusted for age, sex, smoking, genetic ancestry and HLA-DRB1*15:01.

**Table 3:** Matched pairs analysis: Odds Ratios of Multiple Sclerosis by Threshold Values of Serum 25–Hydroxyvitamin D among Blacks, Hispanics and Whites.

25OHD	Blacks			Hispanics			Whites		
nmol/L	Cases *n*	Controls *n*	OR (95% CI)	Cases *n*	Control *n*	OR (95% CI)	Cases *n*	Controls *n*	OR (95% CI)
<50.0	60	65	ref	69	62	ref	61	26	ref
50.0–75.0	20	36	0.54 (0.26–1.10)	65	76	0.76 (0.47–1.26)	91	104	0.30 (0.16–0.55)
>75.0	29	15	2.03 (0.92–4.47)	31	34	0.75 (0.40–1.41)	85	111	0.27 (0.15–0.50)

Presented adjusted odds ratios (OR) and 95% confidence intervals (95% CI) of the association between pre-specified 25OHD threshold values and MS/CIS among blacks, Hispanics and Whites. The 25-hydroxyvitamin D values of cases are deseasonalized based on the matched control values. OR are adjusted for age, smoking and BMI.

## Appendix A

### A.1. Supplementary Methods

#### A.1.1. Study Population, Case and Control Identification and Recruitment

KPSC provides comprehensive health care to ~20% of the population in the geographic area it serves [14]. To identify potentially eligible cases, we searched the complete electronic health record (EHR) electronically monthly for first mention of ICD-9 diagnostic codes for MS or CIS 1 January 2011–31 December of 2014. Diagnoses were confirmed by an MS specialist (ALG) after complete EHR review according to revised McDonald criteria for MS [17] and consensus definitions for idiopathic transverse myelitis (TM) [18,19]. All diagnoses of optic neuritis (ON) were confirmed by ophthalmologists. Symptom onset date and date of diagnosis were validated by structured interview. Patients who met diagnostic criteria for neuromyelitis optica spectrum disorder [43] were excluded. Due to resource limitations and because the primary goal was to study non–white populations, recruitment of white cases was stopped after the target of *n* = 250 had been reached. Study participants of Asian/Pacific Islander (56 participants) and Multiple (2 participants) races were excluded from the analyses due to small sample size. We plan on examining Asian/Pacific Islanders separately once we have recruited sufficient number of participants as we expect the effects of vitamin D and sun exposure on the risk of MS to differ from blacks and whites.

There are slightly more controls in the MS Sunshine Study than cases primarily because sometimes the race/ethnicity indicated in the EHR did not match the participant’s self-reported race/ethnicity. This mis-match between the EHR and self-reported race/ethnicity occurred primarily with white, Hispanics and white, non-Hispanics. To identify eligible controls matched to cases on race/ethnicity, race/ethnicity was initially pulled electronically from the EHR. If, during the structured in-person interview, the control reported a different race/ethnicity than their respective case, another control was recruited for the case until a true racial/ethnic match was completed. Asians (total cases and controls *n* = 57) and people of multiple races (*n* = 2) were recruited but not included in the analyses due to small sample.

The number and proportion of study participants that had 25OHD levels available in their complete electronic health record from prior to symptom onset/index date are as follows: blacks, 17 (14.7%) of cases and 14 (10.7%) of controls; Hispanics, 17 (9.3%) cases, 17 (8.6%) controls; and whites, 20 (8.1%) cases, and 21 (7.9%) of controls.

#### A.1.2. Genotyping

The genotyping data was available on 1159 (97.6%) of the 1187 black, Hispanic or white participants who had completed the study protocol by 6/2/2015. Eighteen participants were excluded due to missing phenotype data as follows: 5 due to missing serum 25(OH)D values; 11 for missing cumulative UVR data; and 2 for missing smoking status. The final analysis cohort included 1141(96.1%) participants: 247 Blacks (116 cases/131 controls), 380 Hispanics (183 cases/197 controls) and 514 whites (247 cases/267 controls).

These participants were genotyped successfully for 697,895 SNPs using Illumina’s HumanOmniExpressExome v1.2. DNA Analysis BeadChips were produced by the Vincent J. Coates Genomics Sequencing Laboratory (GSL) at the University of California, Berkeley. DNA samples were quantitated using the Nanodrop ND–1000 and subsequently normalized and plated for processing. The samples were processed using the Illumina Infinium HD Assay Super protocol. DNA samples were denatured, neutralized, and prepared for amplification. The amplified product was then fragmented, precipitated, and collected by centrifugation. The precipitated DNA was resuspended in hybridization buffer and subsequently hybridized to a beadchip. The beadchip was then prepared for extension and staining. Once the assay was completed, the chips were dried and placed on the scanner for data collection. Data was then QC’d using Genome Studio and Plink.

From Within GenomeStudio, various QC measures were checked including call rates, sex discrepancies, reproducibility and heritability of replicates and CEPH control trios, as well as performance of internal Illumina controls. Any samples with the above discrepancies were noted down. Samples with call rates of less than 90% or less than 99% were also noted. In addition, the following data from PLINK1.07 [21] were filtered: N/percent of SNPs with call-rate < 90%, N/percent of SNPs with Hardy Weinberg (all samples) *p*-value < 0.000001, N/percent of SNPs with MAF < 0.01.

A summary of Control target intensities was then created to evaluate the performance of Illumina’s internal controls. Illumina includes several control targets for the purpose of QC at various stages. The Staining, Extension, Target Removal, and Hybridization controls are all sample-independent measures that show the performance of the assay. The Stringency, Non-Specific Binding and Non–Polymorphic controls are all sample-dependent controls.

We performed sex check and tested the deviation from Hardy-Weinberg equilibrium within racial/ethnic group on SNPs using PLINK 1.07.

#### A.1.3. Deseasonalized 25OHD Values for Other Analyses

For analyses that include unmatched cases and controls, 25OHD levels were deseasonalized for both cases and controls and did not result in significantly different findings. Seasons were combined into 2 seasons, winter (October–March) and summer (April–September) based on the inflection points of the best-fit polynomial regression (quadratic function) of the monthly variation of 25OHD values among controls. This is consistent with Southern California’s annual fluctuation in temperatures and daylight hours. We did not detect significant variation between fall and winter or spring and summer.

#### A.1.4. Cumulative Lifetime UVR

Cumulative lifetime UVR for each participant was calculated using previously validated methods [2]. Average monthly estimates derived by linking latitude and longitude of residence with satellite-derived ground level estimates of ambient UVR multiplied by proportion of time in the sun during summer were summed from age 6 years to symptom onset/index date. Specifically, we used the proportion of self-reported hours outside in the sun for weekends (26 days) and holidays (14 days) in the season (summer: (sun time in hour/12) × 40 to calculate the personal cumulative leisure-time UVR dose in summer for each participant. The leisure-time UVR dose was calculated as ambient UV *x* proportion of day in the sun, then summed over the relevant period, from 6 years of age until symptom onset/index date.

#### A.1.5. UVR 12 Months Prior to Symptom Onset/Index Date and 25OHD

Multivariable linear regression was used to examine the association of UVR during summer and winter 12 months prior to symptom onset/index and log–transformed 25OHD levels including age at index date with sex, BMI and smoking as covariates. We used the proportion of self-reported hours outside in the sun for weekends (26 days) and holidays (14 days) in the season (summer: (sun time in hour/12) × 40 and winter (sun time in hour /8) × 40) to calculate the personal leisure-time UVR dose in summer and winter 12 months prior to symptom onset/index date for each participant.

#### A.1.6. Deseasonalized 25OHD Values for Matched Pairs Analyses

The circulating 25(OH)D level varies over the year; it tends to be higher in summer than in winter, due to the difference in sun exposure habits and ultraviolet intensity. The vitamin D level for case and matched control could be measured at different specific time point. Thus, is important to adjust for the seasonal fluctuation in the measurement of the vitamin D levels. Serum 25OHD values were deseasonalized by fitting a polynomial regression in controls within each race/ethnicity [20]. A quadratic function provided the best fit of the values across weeks within a calendar year. The controls’ 25OHD levels were adjusted by using the fitted curve to the 25OHD levels at the blood collection date of the matched cases and adding individual residual from the model fit (adjusted vitamin D = predicted vitamin D + residual). The cases’ 25OHD levels were unchanged to allow for direct comparison with previous studies [2]. The formulas are as follows:

1. Blacks: *y* = 16.68 + 0.51 × *x* − 0.03 × *x*^2^ + yresid
2. Hispanics: *y* = 19.75 + 0.06 × *x* − 0.02 × *x*^2^ + yresid
3. Whites: *y* = 24.11 + 0.29 × *x* − 0.08 × *x*^2^ + yresid

*y*: the seasonal adjusted 25OHD level
*x*: 1st blood collect date in week
yresid: residual 25(OH)D level

#### A.1.7. Matched Pairs Analyses

To allow for direct comparison with previous studies, conditional logistic regression was used to estimate the OR and 95% CI according to the IOM 25OHD thresholds. Due to the small number of participants with severe vitamin D deficiency (≤30 nmol/L) this was combined with the ≤50 nmol/L group. The conditional logistic regression analysis resulted in eliminating a total of 113 unmatched participants (25 blacks, 44 Hispanics and 44 whites).

### A.2. Supplementary Results

To assess for participation bias smoking status was identified from the electronic health record and age was defined as age upon becoming eligible for recruitment for study participants and those who declined participation. All participants as of 31 December 2015 are included in this table (Table A1) including those recruited after the cut–off inclusion for the analyses in the manuscript of 2 June 2015 as well as those with some missingness precluding inclusion in analyses.

**Table A1:** Participation rates and comparison of participating and declined participants.

Selected Characteristics* According to Participation or Non–Participation (Declined)								
		Participating Cases	Declined Case	*p*-Value	Participating Controls	Declined Controls	*p*-Value	*p*–Value Declined Cases vs. Controls
Blacks		*n* = 126	*n* = 40		*n* = 142	*n* = 143		
	age, mean (SD) years	40.7 (12.7)	42.6 (13.1)	0.41	41.0 (12.8)	42.8 (12.7)	0.22	0.91
	female, *n* (%)	99 (78.6)	25 (62.5)	0.04	109 (76.8)	108 (75.5)	0.81	0.10
	obesity, *n* (%)	54 (42.9)	18 (45.0)	0.81	65 (45.8)	68 (48.9)	0.60	0.66
	BMI, mean SD)	29.8 (7.2)	30.4 (6.8)	0.68	30.8 (7.7)	31.1 (7.9)	0.75	0.57
	participationrate	75.9%			49.8%			
Hispanics		*n* = 192	*n* = 79		*n* = 207	*n* = 219		
	age, mean(SD) years	34.5 (10.8)	37.5 (14.3)	0.10	34.2 (10.9)	37.4 (13.2)	0.01	0.96
	female, *n* (%)	138 (71.9)	50 (63.3)	0.16	152 (73.4)	141 (64.4)	0.04	0.86
	obesity, *n* (%)	79 (41.2)	24 (30.8)	0.11	84 (41.0)	72 (33.0)	0.09	0.72
	BMI, mean (SD)	29.6 (6.6)	29.1 (7.3)	0.58	29.8 (7.1)	28.3 (5.9)	0.02	0.43
	participation rate	70.9%			48.6%			
Whites		*n* = 252	*n* = 86		*n* = 274	*n* = 315		
	age, mean(SD) years	42.5 (12.3)	41.9 (13.5)	0.71	42.3 (12.4)	46.3 (12.0)	<0.01	<0.01
	female, *n* (%)	168 (66.7)	56 (65.1)	0.79	179 (65.)	181 (57.5)	0.05	0.20
	obesity, *n* (%)	79 (31.4)	33 (38.4)	0.23	89 (32.7)	96 (30.6)	0.58	0.17
	BMI, mean (SD)	28.5 (6.8)	28.5 (6.7)	0.98	28.6 (6.9)	28.1 (6.5)	0.37	0.54
	participation rate	74.6%			46.5%			

*: at or closest to the time of eligibility for the study; SD: standard deviation.
